# Bottlenose dolphins are sensitive to human attentional features, including eye functionality

**DOI:** 10.1038/s41598-023-39031-7

**Published:** 2023-08-02

**Authors:** James R. Davies, Elias Garcia-Pelegrin

**Affiliations:** 1 Department of Psychology, University of Cambridge, Cambridge, CB2 3EB UK; 2 Department of Psychology, National University of Singapore, Singapore, 117572 Singapore

**Keywords:** Animal behaviour, Behavioural ecology

## Abstract

The ability to attribute attentional states to other individuals is a highly adaptive socio-cognitive skill and thus may have evolved in many social species. However, whilst humans excel in this ability, even chimpanzees appear to not accurately understand how visual attention works, particularly in regard to the function of eyes. The complex socio-ecological background and socio-cognitive skill-set of bottlenose dolphins (*Tursiops* sp.), alongside the specialised training that captive dolphins typically undergo, make them an especially relevant candidate for an investigation into their sensitivity to human attentional states. Therefore, we tested 8 bottlenose dolphins on an object retrieval task. The dolphins were instructed to fetch an object by a trainer under various attentional state conditions involving the trainer’s eyes and face orientation: ‘not looking’, ‘half looking’, ‘eyes open’, and ‘eyes closed’. As the dolphins showed an increased latency to retrieve the object in conditions where the trainer’s head and eyes cued a lack of attention to the dolphin, particularly when comparing ‘eyes open’ vs ‘eyes closed’ conditions, we demonstrate that dolphins can be sensitive to human attentional features, namely the functionality of eyes. This study supports growing evidence that dolphins possess highly complex cognitive abilities, particularly those in the social domain.

## Introduction

The ability to attribute attentive states to other individuals is a highly valuable socio-cognitive skill and may offer immediate adaptive benefits^1,2^. As well as allowing an individual to know if it is being observed by another individual, attending to a con- or a heterospecific’s attentional cues, i.e., turning to look, can lead to the acquisition of valuable information regarding food resources, predators, or social interactions. Furthermore, the ability to accurately assess what others are attending to is an essential requirement for effective communication, and thus the evolution of such an ability would be advantageous for many social species^3^. This is apparent in humans, as from early infancy children seem to understand the importance of eyes as an indicator of what another individual is attending to. By their first year, children will consistently follow their mother’s gaze^4^ and by two years old, will not use head direction as an indicator of attention if an adult’s eyes are closed or covered^5^.

However, even chimpanzees, our closest living relatives, appear to not accurately understand how visual perception works, particularly in regard to the role of eyes in attention^3,6–8^. When tested on their ability to understand the fundamental conditions underlying the perception of others (relating to body and face orientation as well as the state of the eyes), chimpanzees were unable to attribute attention correctly except for in the most basic condition, in which they chose to beg from either a trainer facing the subject or one who had their back turned^6^. Comparable studies also suggest that multiple non-human primates fail to use eyes as an attentional cue, including chimpanzees^7,8^, rhesus macaques^9,10^, and capuchin monkeys^11,12^. In contrast, other studies report some evidence of the same non-human primates possibly using eyes as an attentional cue. When requesting food from a trainer by pointing to a baited cup, capuchin monkeys looked at the trainer’s face for longer when she returned its gaze than when looking at the ceiling in a first experiment, and looked longer in eyes open vs eyes closed conditions in a second experiment^13^. However, there was no difference in the capuchins’ pointing behaviour between these conditions in either of the experiments. If the monkeys fully understood the role of eyes in attention, it would be expected there would be differences found between these conditions. Furthermore, although Hostetter et al.^3^ found that chimpanzees used more vocalisations when a trainer’s eyes were closed (vs open), the same result was not found when the trainer’s eyes were covered (vs uncovered). This discrepancy therefore reveals failures in the chimpanzees’ ability to interpret attentional states in the presence of (or lack of) eye cues across different experimental contexts. The inconsistent results found in the aforementioned studies may reflect an experimental failure to dissociate different cues given by the trainers. For example, the orientation of the trainer’s body may provide information about their general inclination to give food, whereas the visibility of their face may provide information about their attentional state^14,15^. Unless these information sources are properly separated in the experimental design, the animals’ responses cannot be properly understood.

With socio-ecological backgrounds comparable to that of chimpanzees (including fission–fusion groups, social learning and culture, and complex cooperation^16^) bottlenose dolphins (*Tursiops* sp.) are often placed among the most cognitively advanced species, with abilities rivalling that of the non-human primates^17–24^. As highly social animals, bottlenose dolphin live in pods ranging in size from pairs to around 100 individuals^25^. They demonstrate complex fission–fusion social dynamics^26^ and support one of the most intricate alliance systems documented in non-human animals, including the great apes^27^. Furthermore, dolphins engage in elaborate cooperative hunting strategies^28^, with division of labour and role specialisation^29^, and spread foraging strategies via social networks to form diverse cultures^30,31^. Living within this convoluted social environment may demand a suite of socio-cognitive skills, such as multi-modal individual recognition^32,33^, selective social learning^34^, life-long social memory^35^, episodic-like memory in a social context^36^, and precise behavioural synchronization^37^, as well as abilities that depend on social attention (paying attention to the attention of others)^38^. These include the ability to imitate^21^, the understanding and production of indicative pointing^39–41^, and the capacity to understand the focus of another individual’s gaze^42–45^. It would follow then, that bottlenose dolphins may have evolved a socio-cognitive toolkit that would allow them to successfully use social information to attribute attentional states onto other individuals. Alongside these complex socio-cognitive abilities, captive dolphins, such as the ones tested in this study, are often highly trained animals that routinely perform shows in which their trainers will give them commands of actions to perform (i.e., do a back flip, search for and return a floating object, swim belly up in circles, etc.) in exchange for highly valued rewards. These interactions may prime performing dolphins to be more perceptive of their trainer’s attentional states.

**Figure 1:**
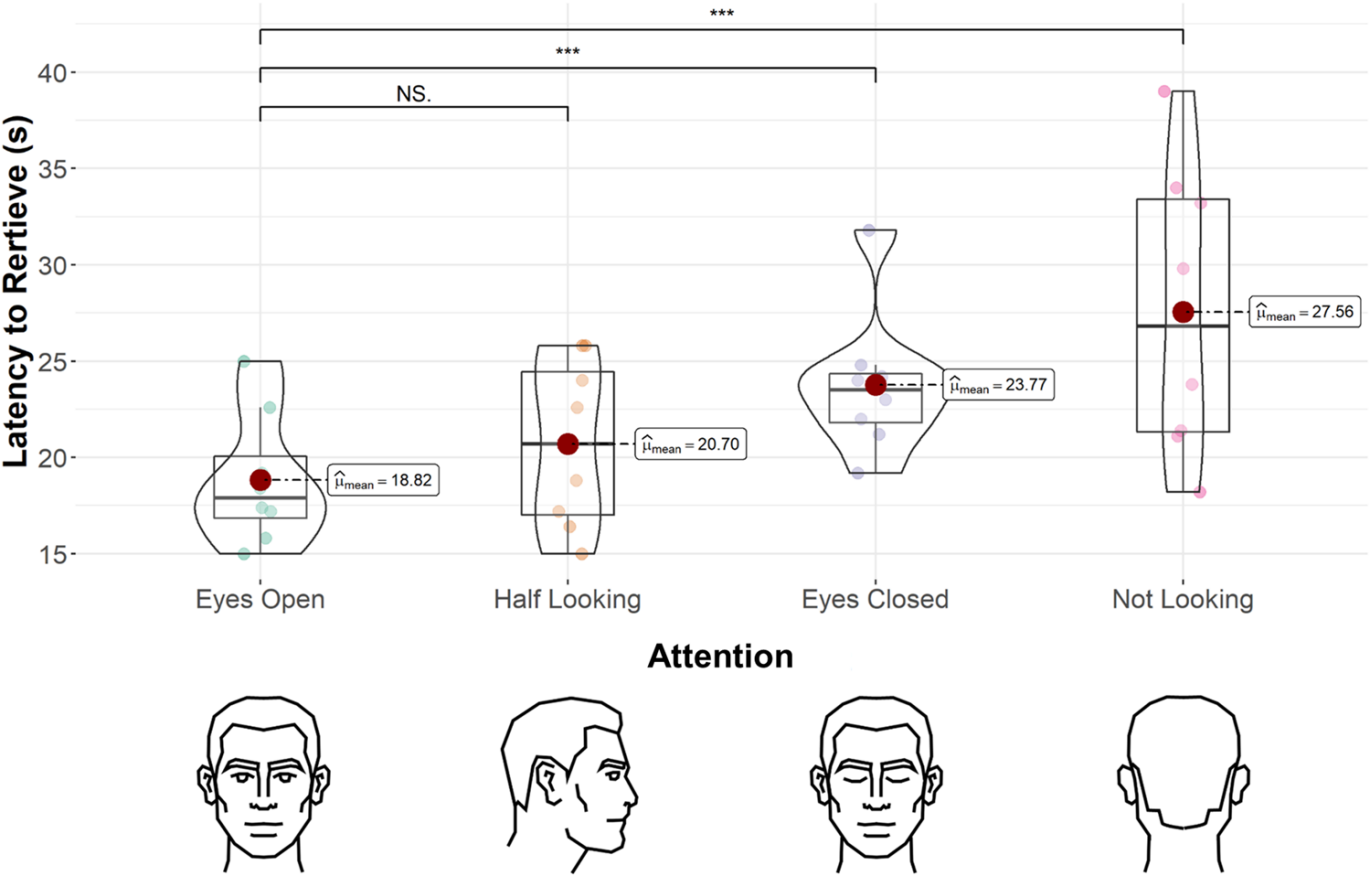
Dolphin’s latency (s) to retrieve the object by attention condition of the trainer. Latency to retrieve the object was measured as the time difference between the moment the command was given to the moment the dolphin raised its body out of the water to give the object to the trainer. Coloured dots show each data point. ***Indicates a p-value of p < 0.05. NS indicates a non-significant p-value.

Therefore, the intricate socio-ecological background and advanced social cognition skill-set of bottlenose dolphins, alongside the highly specialised training that captive dolphins typically undergo, make them a highly relevant candidate for an investigation into their sensitivity to human attentional states, including the functionality of eyes. In this study we tested 8 common bottlenose dolphins (*Tursiops truncatus*) on their sensitivity to human attentional states using an object retrieval task. In this task, the dolphins were commanded by a trainer to retrieve an object from the other side of a pool, whilst varying the visual cues suggestive of the trainer’s attentional levels through four conditions (Fig. 1): ‘eyes open’ (commanding with their head directed towards the dolphin with their eyes open); ‘half looking’ (commanding with their head at a right angle to the dolphin with their eyes open); ‘eyes closed’ (commanding with their head directed towards the dolphin but with their eyes closed); and ‘not looking’ (commanding with their head turned away from the dolphin). To assess the sensitivity of the dolphins to the various attentional state features, we measured the latency to retrieve the object (time difference between the moment the command was given to the moment the dolphin raised its body out of the water to give the object to the trainer). As dolphins can successfully follow human head-directed gaze^43^, we predicted that the dolphins would show a greater latency to retrieve the object in the ‘not looking’ and ‘half looking’ conditions compared to the ‘eyes open’ condition. However, in line with the literature suggesting that non-human primates fail to use eyes as an attentional cue^6–12^, or at best show only partial evidence of responding to eye-related cues^3,13^, as well as the significant physiological contrast in the position of the eyes between dolphins and humans, we also predicted that there would be no significant difference between latencies in the ‘eyes open’ and the ‘eyes closed’ conditions.

## Results and discussion

A generalised linear mixed model (GLMM) revealed that there was a significant effect of attention (χ^2^ = 18.15; n = 8; df = 3; *p* < 0.001; Fig. 1) and no significant effect of trial number (χ^2^ = 4.75; n = 8; df = 3; *p* = 0.19). Between conditions, there was a significant difference in latency to retrieve between ‘eyes open’ and ‘not looking’ (*p* < 0.001; Fig. 1), ‘half looking’ and ‘not looking’ (*p* = 0.002; Fig. 1), and ‘eyes open’ and ‘eyes closed’ (*p* = 0.03; Fig. 1). There was no significant difference between ‘eyes open’ and ‘half looking’ (*p* = 0.39; Fig. 1), ‘half looking’ and ‘eyes closed’ (*p* = 0.21; Fig. 1), and ‘eyes closed’ and ‘not looking’ (*p* = 0.06; Fig. 1).

As the dolphins showed an increased latency to retrieve the object in conditions where the trainer’s head and eyes cued a lack of attention to the dolphin, this suggests that dolphins can be sensitive to human attentional features. As latency in ‘not looking’ conditions differed significantly from ‘half looking’ as well as ‘eyes open’ conditions, this suggests that, in line with our first prediction, the dolphins could use head direction to successfully attribute human attentional states. However, as we found a significant difference between ‘eyes open’ and ‘eyes closed’, contrary to our second prediction, but not between ‘eyes closed’ and ‘not looking’, these results also suggest that the dolphins could successfully use eye cues to attribute human attentional states. That said, we must note that the difference between ‘eyes closed’ and ‘not looking’ conditions approaches significance and thus may likely reach significance with a larger sample. The lack of difference between the ‘half looking’ and both ‘eyes closed’ and ‘eyes open’ conditions may reflect confusion over conflicting head vs eye cues. The dolphins’ ability to successfully attend to eye cues is particularly interesting, considering the inconsistent evidence for this ability in chimpanzees and other non-human primates, as well as the distinct difference in eye placement between humans and dolphins. Therefore, these results, alongside other evidence^21,32–37,39–44^, raise the possibility that bottlenose dolphins possess complex social cognitive abilities.

Whilst this study is the first to evidence that bottlenose dolphins are able to use the functionality of eyes (open or closed) as an indicator of human attentional states, our findings are supported by other research demonstrating that dolphins can follow human gaze and pointing gestures^42–44^, as well as responding to human body orientation as a cue of their attention. For example, in two-object choice tasks, dolphins can successfully follow human-directed gaze and pointing gestures^42,43^, as well as indicating the identity of an object a human is pointing to in addition to its location^44^. However, they were not successful at following gaze cues when only the eyes were used (with the head stationary)^43^, and when the objects were placed at different distances away from them (as opposed to laterally in front of them)^44^. Xitco et al.^41^ show that dolphins pointed (facing in a direction for 2 s or longer) more towards jars containing food when a trainer was facing them compared to when the trainer’s body was turned away from them. Furthermore, they were significantly more likely to leave the testing apparatus during back-turned trials compared to face-forward trials, and rarely pointed during trials where the experimenter swam away after baiting the jars.

However, our inferences about which attentional cues (e.g., body and/or head) used by dolphins differ from comparable studies that independently varied the orientation of these features^46^. For example, in a study in which bottlenose dolphins had to follow gestural commands given by humans with varied attentional states, Tomonaga et al.^46^ found that the behaviour of the dolphins seemed to be controlled by the orientation of the trainer’s body but not their head. However, as Tomonaga et al., only orientated the trainer’s head direction through 90 degrees, representing the equivalent of our ‘half looking’ condition, they may have found an effect of head direction if they had fully rotated the head away from the dolphin, as in our ‘not looking’ condition. Furthermore, the authors argue that as the dolphins were tested in an experimental context very similar to their daily performance training and husbandry, and used the same gestural commands used within a strict positive reinforcement procedure, the dolphins may have ignored the subtle social cues presented by the trainers as they had been trained extensively to follow only the command gestures. In contrast, we tested our dolphins outside of their usual training context, both in a physically separate pool (usually used for veterinarian purposes) as well as differing from their daily performance training schedule in terms of timings, trainers, and the context of the commands (commands are usually given with the trainers full attention). Alternatively, the discrepancy in findings may reflect differences in the behavioural responses measured. Tomonaga et al., tested dolphins on their ability to produce correct responses to commands, whereas we tested our dolphins on their latency to perform a behaviour. Therefore, our analysis was able to detect much more subtle differences in behaviour across conditions, which Tomonaga et al., may not have been able to discern using binary (correct vs incorrect) measures. That said, altering the trainer’s body orientation in Tomonaga et al., necessarily changed what the commands looked like to the dolphins, and so their responses may not have been related to the attention of the trainers, but rather confusion over what command was being given. These results may also be due to differences in the information that body and head direction represent^14,15^. Tempelmann et al.^15^ found that as long as giving food was no longer restricted by body orientation, the orientation of the trainer’s head became the main factor in dictating apes’ behaviour. As the trainer’s body remained in the same position across all conditions in the current study, unlike Tomonaga et al.^46^, head and eye cues seem to have been attended to by our dolphins, which follows the conclusions reported by Tempelmann et al.^15^.

Perhaps a more parsimonious explanation for the increased latency in conditions differing from ‘eyes open’ could be attributed to the dolphins’ expectations of how a command of this nature is typically presented, which may have been contradicted or disrupted. Indeed, it is important to note this sample of dolphins has been extensively trained with the ‘retrieve’ command in a context in which the trainer is giving them their full attention (i.e., head facing the dolphin with their eyes open), and thus the situation experienced by the dolphins in this experiment is novel. Therefore, there is a possibility that the increased latency in conditions differing from ‘eyes open’ represents an expectancy violation due to confusion surrounding the trainer’s behaviour. Due to the limitations associated with the methods of analysis employed in this study, it is not practically possible to separate this possibility independently from the observed behavioural responses exhibited by the dolphins in our sample. However, we deem this prospect unlikely due to the subtle visual differences between ‘eyes open’ and ‘eyes closed’ conditions, and the reported lack of difference between the ‘eyes open’ and ‘half looking’ conditions, as well as the ‘eyes closed’ and ‘not looking’ conditions (although the later relationship was approaching significance (*p* = *0.06*) and would likely be significant in a more powered study). Furthermore, it is crucial to acknowledge that even if such a violation of expectation were to occur in the ‘eyes closed’ condition for the dolphin sample, it would still indicate a significant sensitivity towards the human trainers’ eyes. This sensitivity would remain noteworthy, irrespective of whether it is specifically attributed to the attentional aspects of human vision or not.

Interestingly, whilst our dolphins demonstrated their sensitivity to the factors involved in human visual attention, this ability does not seem to correspond directly to their primary method of communication in nature^47,48^. The ‘cooperative eye hypothesis’ states that human eyes, compared to other primates, allow for particularly visible signals (due to the white sclera and dark pupil) and that we may have evolved this feature in order to facilitate cooperation with conspecifics through gaze following in close-range joint attentional and communicative interactions^49^. Therefore, the ability to attribute attentional states based on visual signals may have been highly selected for in hominin evolution. Dolphins, on the other hand, appear to communicate and interpret their environment primarily through acoustic signals, using high-pitched whistles and echolocation clicks^47,48^. For example, in a cooperative task, dolphins were shown to use vocal signals to facilitate the successful execution of the task, as they were significantly more likely to cooperate successfully when they produced whistles^37^, and were less successful in the presence of distracting anthropogenic noise^50^. Dolphins also appear to use echolocation cues in role-specialised group hunting and produce more whistles during this group hunting strategy than when solo foraging^29^. Furthermore, males within alliances formed for coercive mate guarding, in which males cooperate to control female movements through aggression, use vocal communication to coordinate their movements^51^ and to increase their bond strength^52^ (with direct fitness implications^53^).

Nevertheless, dolphins do appear to somewhat rely on visual information in social interactions and cooperation. Whilst more successful when using whistles in the cooperative task, dolphins were still able to coordinate their behaviour without producing any whistles, and thus, as suggested by the authors, were likely using visual information to succeed in these cases^37^. Furthermore, the coordination of movement, resulting in elaborate synchronous displays, has been demonstrated to play an important role in dolphin social interactions. Most motor synchrony behaviour is witnessed between males within female-consortship alliances^54^, which include joint surfacing, aerial leaps, and underwater turns in different directions (e.g., parallel swimming, swimming in opposite directions, etc.)^26^. These displays are most common in the presence of females, but also likely function in strengthening the alliance^54^. It is highly likely that these synchronous displays rely on visual information to coordinate movement, particularly as this synchrony is remarkably precise, with behaviours separated by just 80–130 ms^54,55^. Moreover, dolphins perform an ‘S-shape’ posture as a signal of aggression in sexual interactions and disciplinary behaviour toward juveniles^47^. Whilst these observations do suggest a role of visual information in dolphin social interactions, there is no evidence of dolphins using conspecific eye-related cues as signals in the wild or captivity. Although Johnson et al.^45^ report evidence of conspecific “gaze-following” in dolphins, this behaviour could be performed through utilising body, head, or movement cues alone, without the use of the eyes, and may also involve vocalisations. Therefore, the ‘cooperative eye hypothesis’ is not sufficient in explaining the behaviour of our dolphins in the current study, as it is unlikely that there has been significant selection in the dolphin lineage to evolve the ability attribute attentional states towards conspecifics based on visual signals.

Consequently, the ability of our dolphins to attend to human attentional cues may lie in their training and subsequent development in captivity. Indeed, such specialised and intense training might have primed performing dolphins to be more perceptive of their trainers’ attentional states, as these would be highly associated to the possibility of the trainer asking for a particular behaviour and acting as a faithful reference point for a dolphin's own behavioural regulation (i.e., if the trainer is not paying attention to me, then there is no need for me to attend to the trainer). Moreover, trainers often direct more than one dolphin simultaneously, and these are usually not commanded equally. For example, one dolphin might be asked to do a back flip whilst the other one is asked to show their flukes. Therefore, the attentional state of the trainer (i.e., is the trainer looking at me or the other dolphin) gives the dolphin a powerful cue to discern what it is being asked to do (and subsequently rewarded for). Perhaps then, whilst not relevant in nature, these dolphins may have learned to use human eyes as a cue reflecting their attentional state in order to accurately interpret and perform various behaviours commanded by their trainers for rewards. In fact, other species with comparable training relationships with humans, such as dogs and horses, have also shown evidence for a sensitivity towards human attentional states^56–62^. This has been argued to be an innate ability facilitated by selective pressures involved in domestication^57^, or instead (although not mutually exclusive) due to learning from repeated experiences with humans over years of training. The later hypothesis is supported by a study testing young domestic horses which reports that although they were successful in using a human trainer’s body orientation as an attentional cue, they could not, in contrast with adult trained horses^61^, use more subtle cues such as their head orientation and eye functionality^62^. Therefore, in at least some species it seems that significant experience is required to develop an ability to attend to subtle human attentional cues.

As all the dolphins in this experiment have been trained to perform behaviours from an early age, having been born and raised in captivity, it would be highly informative to assess the ability of untrained dolphins to attribute human attentional states, whether in the wild or captivity. Similarly, it would also be highly enlightening to assess the attention attribution abilities of dolphins which, although wild and untrained (i.e., to perform in shows), have had long-standing mutualistic interactions with humans, such as the population of bottlenose dolphins living off the coast of Laguna, Brazil^63^. These dolphins regularly cooperate with local artisanal fishers in a century-old practice whereby dolphins shoal fish towards the shore, and experience increased foraging success as a result of the fishers casting their nets in synchrony with the dolphins’ dive cues^64^. As the dolphins only modify their foraging behaviour, which is necessary for them to actually benefit from the interaction, when the fishers respond appropriately to their foraging cues^65^, it may be beneficial for the dolphins to attend to the humans’ attentional features in order to maximize their foraging efficiency and not waste effort on inattentive cooperators. Assessing possible variation in the ability to attribute attentional states onto other individuals (namely humans) across different populations of dolphins with differing life experiences, may reveal whether this ability is shared amongst the entire species, or subject to behavioural/cognitive flexibility and therefore learned through relevant experience.

It could be argued, however, that due to the limited evidence of dolphins relying on visual information (particularly eye-related cues) for social interactions, as well as the anatomical and sensory differences between dolphins and humans, the dolphins in this study had learned that it is more “rewarding” to retrieve the object when the trainer was facing them with their ‘eyes open’ than when they were ‘not looking’, rather than a full understanding of the concept of gaze direction and eye-related attention. However, as the experimental situation (in which the trainer was giving the dolphin anything but full ‘eyes open’ attention) was novel, and that each dolphin only participated in a single trial per condition, they did not have the opportunity to learn this difference through repeated experience. Moreover, the dolphins were always rewarded equally across the conditions, providing they successfully retrieved the object and gave it to the commander, which further casts doubt on the validity of this argument.

Furthermore, whilst this study only measured the behavioural response of the dolphins through their physical behaviour (latency to retrieve the object), designing a similar task in a context where vocalisations could be used (i.e., to attain the trainers attention when they are not looking, or their eyes are closed) would be highly informative to our understanding of attentional attribution in dolphins, as vocalisations appear to be much more important in bottlenose dolphin social interactions, as detailed above. In addition, whilst relatively large for comparative psychology experiments, our sample size of 8 dolphins is fairly small for measuring variation in latency. Although we were able detect significant variation between conditions, even with this small sample size, a replication with a larger sample size would nevertheless be highly informative to validate the robustness of our results. Furthermore, as the behavioural response measure of ‘latency to retrieve an object’ is somewhat abstract and could be controlled by other unmeasured factors that do not involve attentional state attribution, such as expectancy violation, investigating whether the attentional state of the trainer impacts the dolphins’ behavioural responses in other tasks, for example performing trained performance related behaviours, e.g., a backflip, may corroborate the findings of this study (i.e., the dolphin may perform a behaviour less successfully when the trainer cues a lack of attention).

Nevertheless, this study adds valuable insight regarding the question of how non-human animals perceive and understand social information, which is a topic predominantly studied using chimpanzees and other non-human primates. As we build on evidence suggesting that dolphins may be able to attribute attentional states to humans^41^, and demonstrate for the first time the successful use of head orientation and eye functionality-related attentional cues, this discovery opens new avenues of research into dolphin social cognition as well as inter-species cooperation and communication.

## Methods

### Subjects and housing

8 dolphins (3 females; 6–29 years old) participated in this study. This sample was comprised of all the dolphins available for testing (i.e., not calves and in full health). Dolphins were individually identified using their distinctive physical characteristics, such as facial differences and tooth rake patterns. The dolphins were grouped into three pods, housed in three adjacent pools (pool 1 = 2581 m^3^; pool 2 = 1840 m^3^; pool 3 = 1840 m^3^) at Zoomarine Italia (Torvaianica, Italy). This arrangement was already in place before the current experiment and was in line with individual social and breeding requirements. The pools were connected via gates, which also connected to an additional two veterinarian pools, one of which was used for this study (1020 m^3^). The experimental pool was not typically used for training purposes. Overall, the pools totalled to 7667 m^3^. Each dolphin that participated in this study was born and raised in captivity. The dolphins had previously participated in other research using the same commands^36^. Zoomarine trains their dolphins to perform different behaviours for various reasons, including medical care and zoo performances, as in common in zoological settings^66^. This is done when the dolphins are young, through positive reinforcement based operant conditioning and social learning. For testing, the dolphins were individually isolated in the experimental pool, when possible, but if not (e.g., when testing a cow with a young calf), the calf was kept at the other side of the pool by a trainer.

### Procedures

The project was approved by the University of Cambridge Animal Welfare Ethical Review Body and was conducted under a university non-regulated procedure licence (OS2022/01) and all methods were performed in accordance with relevant guidelines and regulations. This study is reported in accordance with ARRIVE guidelines. Dolphins were tested on their sensitivity to human attentional cues using an object retrieval task. Before the onset of the current study, all dolphins were trained to retrieve an object floating in the water by Zoomarine staff, using a specific command. This command consisted of two consecutive hand motions pointing with a closed fist (index finger facing upwards) in the direction of the object. Before any commands were given, in normal Zoomarine training and for this study, individuals are encouraged to face the trainer and attend to the subsequent command (using other trained commands: a flat palm facing either up or down, used to encourage the dolphin to approach and to signal to the dolphin that they are about to receive a command, respectively). Commands were only given once the dolphin was positioned directly in front of the commanding platform (the dolphins were trained to wait for the command in this position) and was facing the trainer (who was informed by other trainers in conditions when they could not see the dolphin). In test trials, the dolphins were commanded by a trainer to retrieve an object from the other side of the pool, whilst varying the visual cues suggestive of the trainer’s attentional levels through four conditions (Fig. 1): ‘eyes open’ (commanding with their head directed towards the dolphin with their eyes open); ‘half looking’ (commanding with their head at a right angle to the dolphin with their eyes open); ‘eyes closed’ (commanding with their head directed towards the dolphin but with their eyes closed); and ‘not looking’ (commanding with their head turned away from the dolphin). In all conditions, the trainer’s body remained perpendicular to the dolphin (so that the head could be swivelled in either direction without moving the body). The ‘eyes open’ condition can be thought of as a control condition, as it is the context in which commands are usually given. Each dolphin received one trial per condition in a randomised order (using Excel function = Rand()) to control for any effect of trial order (all dolphins had a unique trial order). In order to standardise the location of the object in the pool, it was temporarily held in position by a second trainer until the dolphin obtained it. To assess the sensitivity of the dolphins to the various attentional states, we measured the latency to retrieve the object (time difference between the moment the command was given to the moment the dolphin raised its body out of the water to give the object to the trainer).

### Analysis

We video recorded all trials and coded all videos using Solomon Coder^67^. We second coded 20% of videos and inter-rater reliability was strong: (Cohen’s Kappa = 0.8). The second coder was blinded to the study’s aim whilst coding. We conducted a generalised linear mixed model (GLMM) to test whether condition (attentional input) influenced the dolphin’s latency to retrieve the object, with dolphin as a random effect and attention condition and trial number as fixed effects, using likelihood ratio test (drop1() function) and Tukey post hoc comparisons (package multcomp^68^, function glht()). To check our model’s assumptions, we used the DHARMa package^69^. Our model did not fail to converge and exhibited a confidence interval of 97.5%. The assumption checks of our model evidenced no deviation from the expected distribution but showed some quantile deviations of the residuals against the predicted values.

## Data Availability

The datasets generated during and/or analysed during the current study are available from the corresponding author on reasonable request.
